# Effect of different drying temperature settings on the color characteristics of Tencha

**DOI:** 10.1016/j.fochx.2024.101963

**Published:** 2024-11-05

**Authors:** Ya-Lin Mao, Jie-Qiong Wang, Fang Wang, Qing-Qing Cao, Jun-Feng Yin, Yong-Quan Xu

**Affiliations:** a Modern Agricultural institute, Jiaxing Vocational & Technical College, Jiaxing 314036, China; b Tea Research Institute Chinese Academy of Agricultural Sciences, Key Laboratory of Biology, Genetics and Breeding of Special Economic Animals and Plants, Ministry of Agriculture and Rural Affairs, Hangzhou 310008, China

**Keywords:** Tencha, Drying, Temperatures, Color, Pigments, Chlorophylls

## Abstract

Color is critical factor in the commercialization of Matcha. In this study, sensory evaluation, color difference analysis, as well as targeted and non-targeted analyses were employed to investigate the impact of different drying temperature settings on the color characteristics of Tencha. The findings revealed that compared to a single drying temperature setting, a two-stage or multi-stage drying process more effectively preserved the color quality of Tencha. Specifically, a setting involving an initial period of high-temperature drying followed by low-temperature drying (samples T_6, T_7, T_10, and T_13) resulted in superior tea color quality, characterized by higher chlorophyll content and lower levels of lutein and *β*-carotene. Chemometric analysis identified chlorophylls and their derivatives (chlorophyll *a*/b, pheophytin a/b, pyropheophytin a/b) as the key factors influencing Tencha's color. These results can provide valuable insights for optimizing tea processing methods to enhance quality.

## Highlights

• Metabolomics was used to characterize the effect of drying on the color of Tencha.
• Chlorophyll and its derivatives were the key influences on the color of Tencha.
• Short-term high-temperature and then low-temperature can maintain the green color of Tencha.

## Graphical abstract

**Figure:**
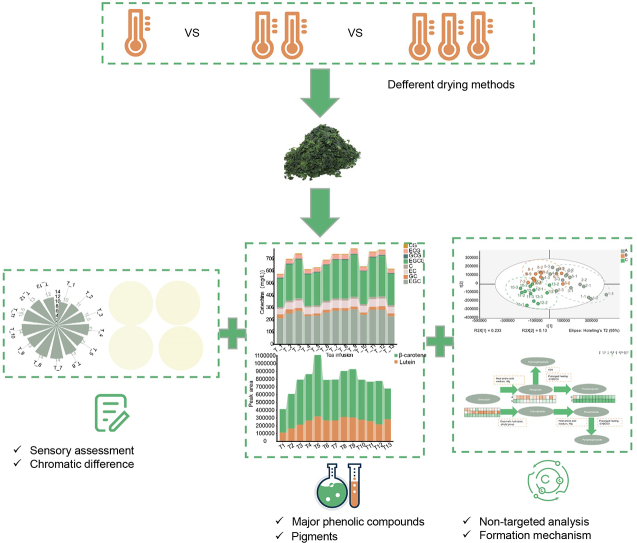

## 1 Introduction

Matcha is a form of powdered steamed green tea derived from grinding Tencha leaves (Tan et al., 2019). Tencha, specifically made for Matcha, serves as its precursor.

Unlike traditional green tea, which is brewed and strained, Matcha is consumed directly in the form of ultra-fine powder and incorporated into beverages and foods. In addition, Matcha is incorporated into everyday products such as toothpastes, facial mask and so on (Wang, Yin, & Xie, 2019). The quality assessment of Matcha differs significantly from that of other green teas. Color is a critical parameter in the sensory evaluation of Matcha (Kim et al., 2020; Xiao, Tao, & Liu, 2021). In China, the color of the tea leaves and its infusion constitutes 40 % and 15 % of the sensory evaluation criteria for Matcha or Tencha, respectively (Mao, Wang, Yin, & Xu, 2020). This aspect is pivotal not for the commercial value but also for consumer acceptance of Matcha and its derived products.

The color of green tea is predominantly influenced by lipid-soluble pigments, mainly including chlorophyll *a* (Chla), chlorophyll *b* (Chlb), lutein and *β*-carotene, play the leading roles (Li et al., 2021). During tea production, these pigments are susceptible to degradation by light, oxygen and heat, leading to formation of pigment derivatives such as pheophytin a (Phe-a) and pheophytin b (Phe-b) (Gaur, Shivhare, & Ahmed, 2006). These derivatives can adversely affect the tea's color quality (Li, Jin, Sun, Ye, & Liu, 2019). According to Dai, Xiao, Zhu, and Hua (2020), the color of green tea infusion was influenced not only by water-soluble pigments but also by lipid-soluble pigments, which were primarily composed of a small amount of chlorophyll and flavonoids. Therefore, the levels of chlorophylls and carotenoids are crucial determinant of Matcha's coloration and overall quality. A previous study highlighted that near-infrared (NIR) spectroscopy was a viable method for the rapid quantification and monitoring of chlorophyll during the processing of Matcha (Liu et al., 2023). However, there is limited research on the combined application of sensory evaluation with targeted and untargeted characterization for assessing the color quality of Tencha.

To achieve a greener Matcha, several strategies have been implemented, such as shading tea trees before harvest to minimize photosynthesis and preserve chlorophylls levels (Tomohito, Hideki, Akiko, & Yuhei, 2018; Wang, Park, Chung, Baik, & Park, 2004; Zhu, Liu, Cheng, Li, & Liu, 2023). Additionally, techniques like short-time and high-temperature steaming fixation and low temperature grinding are employed to minimize chlorophyll degradation. The drying process must rapidly reduce moisture content without compromising the quality of active tea components (Engelhardt, Bahar, & Delker, 2023; Lenka, Jana, Dominika, & Klaudia, 2015). It is worth noting that drying significantly affected the color of tea (Lee, Hwang, Kang, & Choung, 2015; Martina, Bohuslava, Ivan, Jana, & Petra, 2014). Factors such as temperature, relative humidity, and drying duration contributed to color degradation in tea leaves (Natthawuddhi & Yukiharu, 2019; Sahar, Mehdi, & Sayed, 2016). Despite the importance of drying in determining Tencha's color quality, previous research has predominantly focused on changes in volatile and non-volatile compounds during drying (Liu, Rong, Chen, & Ouyang, 2024; Xiao et al., 2022). There is a gap in understanding the color changes and the mechanisms affecting Tencha under various drying temperature settings.

Therefore, the scope of the present study is to systematically elucidate the impact of different drying temperatures and times on the color characteristics of Tencha, and which compounds and their concentrations are pivotal in determining Tencha's color. This knowledge could be crucial for improving tea processing methods and enhancing the objectivity of tea quality evaluation.

## 2 Materials and methods

### 2.1 Chemicals and regents

The (−)-catechin (C), (−)-epicatechin (EC), (−)-catechin gallate (CG), (−)-epigallocatechin (EGC), (−)-gallocatechin (GC), (−)-epicatechin gallate (ECG), (−)-gallocatechin gallate (GCG), (−)-epigallocatechin gallate (EGCG), Caffeine [≥99 %, high-performance liquid chromatography (HPLC)], chlorophyll *b* (≥90 %) and *β*-carotene (≥95 %) were purchased from Sigma-Aldrich (Shanghai, China). Phytoxanthin (HPLC, ≥90 %) was purchased from J&K Scientific Co., Ltd., (Beijing, China). Chlorophyll a (≥90 %), pheophytin a (≥90 %) and pheophytin b (≥90 %) were purchased from Wako Pure Chemical Industries, Ltd. (Japan). Acetonitrile (ACN, HPLC grade), methanol (MeOH, HPLC grade) and glacial acetic acid (HPLC, ≥99.9 %) were purchased from Merck (Darmstadt, Germany). Ethanol (GR grade), chloroform (GR grade) and acetone (GR grade) were obtained from Sinopharm Group Chemical Reagent Co., Ltd. (Shanghai, China). Pure water used in this experiment was purchased from Hangzhou Wahaha Group Co., Ltd. (Hangzhou, China).

### 2.2 Tencha manufacturing and sample collection

Fresh tea leaves, consisting of one bud and three or four mature leaves from *Camellia sinensis* (L.) O. Kuntze cv. Yabukita, were harvested on May 28, 2020, and provided by Shaoxing Royal tea village Co. Ltd. (Zhejiang Province, China). Prior to harvest, the leaves were shaded for approximately 21 days to enhance chlorophyll retention. Following harvest, the leaves were fixed by steaming (100 °C, 20 s) to halt enzymatic activity, and were then spread out and cooled immediately. Subsequently, the cooling leaves were subjected to different drying procedures to produce Tencha samples from T_1 to T_13, and the moisture content of the samples was less than 5 %, as detailed in Table S1.

### 2.3 Sensory evaluation

The color characteristics of Tencha and its infusion was evaluated by a trained panel of five panelists (comprising three men and two women, aged 26 to 50) from the Tea Research Institute of the Chinese Academy of Agricultural Sciences. All panelists were certified in tea quality evaluation by the Tea Scientific Society of China and possessed considerable expertise in sensory evaluation. Prior to conducting the sensory evaluation, each panelist provided written informed consent, and the study was approved by the Tea Research Institute of the Chinese Academy of Agricultural Sciences. Firstly, Tencha was placed in an evaluation plate, and its color was observed and rated on a scale of 0–40. Results were recorded systematically. Subsequently, the color of tea infusion was evaluated. For this purpose, a special sieve (obtained from Japan) was used in conjunction with a matching white porcelain bowl (obtained from Japan). A 3 g Tencha samples was placed into the sieve, which was immersed in boiling water (about 175–180 mL) for 1.5 min. Following the steeping period, the sieve was removed, and the color of tea infusions was evaluated and scored on a scale of 0 to 15 (Mao et al., 2020). The remaining tea infusions were used for subsequent physical and chemical testing.

### 2.4 Analysis of chromatic difference

The chromatic difference of Tencha and their tea infusions were measured using a Minolta CM-600d and CT-310 automatic colorimeter (Konica Minolta China, Shanghai, China). Tencha was placed in a square white tea evaluation tray, and measurements were taken from an average of five different points to obtain a single measurement value. The chromatic difference analysis between Tencha and its infusions was performed through three replicates of the experiments. The color parameters evaluated included L* (lightness component, where 0 represented black, 100 represented white), a* (red-green component, with positive values indicating red and negative values indicating green), and b* (yellow-blue component, with positive values indicating yellow and negative values indicating blue). Each parameter was measured in quintuplicate for each sample. The hue of the color was determined by the ratio of b*/a*, where a larger absolute value of b*/a* indicated a lower degree of green (Mao et al., 2020). The spectrophotometer was calibrated with a D65 light source and set to an observer angle of 10°. For color visualization, the color space conversion function at https://www.qtccolor.com/ was utilized (Wang et al., 2023).

### 2.5 Analysis of non-volatile components of Tencha

#### 2.5.1 Analysis of catechins and caffeine

Catechins and caffeine in tea infusions were analyzed using HPLC (Shimadzu LC-2010A HPLC system, Shimadzu Corporation, Kyoto, Japan). The chromatographic separation was performed with a Symmetry C18 column (5 μm, 4.6 mm × 250 mm) maintained at a temperature of 40 °C. Detection was conducted using an SPD-20 A UV/VIS detector set to a wavelength was 280 nm. Mobile phase A consisted of H₂O with 2 % acetic acid, and mobile phase B was ACN. The elution gradient was programmed as follows: the percentage phase B started with 6.5 % and was linearly increased to 15 % over 16 min, to 25 % over 25 min, and maintained at 25 % for 0.5 min, then decreased to 6.5 % B at 30 min, followed by equilibration with 6.5 % for 5 min. The total run time was 35 min with a flow rate of 1 mL/min. Prior to injection, tea infusions were filtered through a 0.45 μm Millipore filter. The injection volume was 10 μL (Wang et al., 2024). Each sample was analyzed in triplicate.

#### 2.5.2 Analysis of tea pigments

The pigments in Tencha were extracted according to previous study (Cao, Chen, Xu, & Yin, 2020). Briefly, 50 g of Tencha was milled 30 s under dark conditions. A 500 ± 10 mg aliquot of the tea powder was extracted with 5 mL 80 % glacial acetone (prepared by mixing 80 % acetone with water storing it at 4 °C overnight; mixture should be kept on ice during use) for 2 h. The extracting solution was then centrifuged at 5000 rpm for 10 min. The supernatant was transferred to a 25 mL brown volumetric flask. The extraction procedure was repeated up to 5 times or until the tea powder appeared completely white. The extraction process was conducted in darkness with intermittent shaking for 0.5 h per extraction, and the final pigment extract was brought to a constant volume with 80 % glacial acetone.

The pigments analysis was performed using an Acquity ultra performance liquid chromatography H-Class system (UPLC, Waters) as previously described (Cao et al., 2020). Separation was achieved with an ACQUITY UPLC HSS T_3 column (2.1 × 100 mm, 1.8 μm, Waters) at a column temperature of 35 °C. Detection was carried out over a wavelength range of 210 to 600 nm. The gradient elution was conducted with mobile phase A (ACN: acetic acid: water = 3: 0.5: 96.5, *v/v*) and B (ACN: methanol: chloroform = 75:15:10, *v/v*) at a flow rate of 0.413 mL/ min. The elution program started with 80 % B, with the following elution conditions: 0–4.53 min, from 80 % B to 100 % B; 4.53–7.93 min, 100 % B; 7.93–13 min, from 100 % B to 80 % B.

#### 2.5.3 Chlorophylls derivatives analysis based on ultrahigh-performance liquid chromatography-high resolution mass spectrometer (UHPLC-HRMS)

Tencha lipid profiling analysis was performed using UHPLC-HRMS. The mass spectrometry analysis was conducted with a Q Exactive system (Thermo Fisher Scientific, Rockford, IL, USA). Briefly, tea lipid extracts were separated on an ACQUITY UPLC HSS T_3 column (2.1 mm × 100 mm, 1.8 μm, Waters). The mobile phase composition was as follows: mobile phase A consisted of acetonitrile with 40 % water and 10 mM ammonium acetate, while mobile phase B comprised isopropanol with 10 % acetonitrile and 10 mM ammonium acetate. The elution gradient was programmed as follows: 0–2 min at 32 % B; 2–4 min, ramped to 60 % B; 4–13 min, increased to 97 % B and maintained for 4 min; thereafter decreased to 32 % B in 0.1 min and equilibrated for 2.9 min prior to the next injection. The total run time was 20 min (Li et al., 2017). Data acquisition was performed in electrospray ionization (ESI) positive mode with a capillary voltage of 3 kV. The source temperature and desolvation temperature were set at 120 °C and 450 °C, respectively. The cone gas flow and desolvation gas flow were maintained at 50 and 800 L/min, respectively. The mass scan range was set from mass-to-charge ratio (*m/z*) 200 to 1200.

Each sample was analyzed in triplicate. The quality control (QC) sample, prepared by combining equal volumes of all tea samples, was used to monitor stability and repeatability throughout the analysis. After data acquisition, the Compound Discoverer software was employed for ion extraction from the raw data. Subsequently, data filtering was conducted, the remaining ions were used for further Principal Components analysis (PCA), Partial Least-Squares Discriminant Analysis (PLS-DA) and heatmap analysis to distinguish different sample types and identify significant differences in substances (Fu, Wang, Chen, Wang, & Xu, 2020; Wang et al., 2022).

### 2.6 Statistical analysis

All results were expressed as mean ± standard deviation (SD) from three replicates. Statistical analysis to determine significant differences between means was performed using one-way ANOVA with SPSS software (Version 20.0). Graphs were generated using Origin software (version 2018). The PCA and PLS-DA were conducted using SIMCA-P 13.0 software.

## 3 Results and discussion

### 3.1 Color characteristics of Tencha using single-temperature drying procedure

#### 3.1.1 Color sensory quality and chromatic difference analysis

**Fig. 1:**
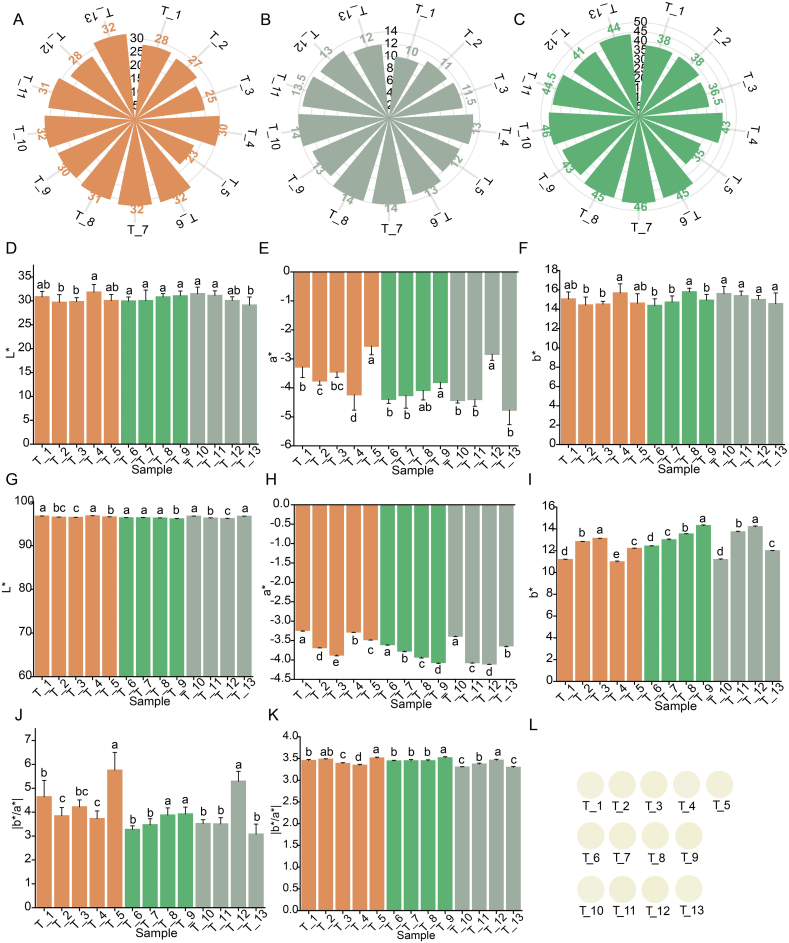
Color characterization of tencha and its brewed tea infusion under different drying settings temperatures. **A-C).** Sensory scores for dry tea (A), tea infusion (B), and overall color (C). **D—F)**. Color difference parameter L* (D), a*(E), b* (F) for dry tea. **G-I).** color difference parameter L* (G), a* (H), b* (I) for tea infusion. **J-K)**. Values of |b*/a*| for dry tea (J) and tea infusion (K). **L).** Visualization of tea infusion.

Distinct color characteristics were observed in Tencha samples T_1 to T_5, which were subjected to different single-temperature drying procedures. The color scores of dry teas, tea infusion and the total scores were highest for T_4, with values of 30, 13 and 43, respectively, whereas T_5 exhibited lower scores of 23, 12 and 35 (Fig. 1A-C). The drying temperatures applied to samples T_1 to T_5 were progressively reduced (Table S1), suggesting that T_5 processed at lower temperatures did not exhibit superior color quality. Overall, the Tencha samples with different drying temperature settings showed variations in color parameters. Sample T_4 had significantly higher values of L* and b* (*p* ≤ 0.5), and lower value of a* and |b*/a*| compared to the other samples. In contrast, T_5 had lower values of L* and b* values, and significantly higher value of a* and |b*/a*| (*p* ≤ 0.5) (Fig. 1D-F, J). These findings indicated that T_4 had a higher brightness and a distinctive yellowish-green color, whereas T_5 had a lower brightness and a distinctive greenish-yellow color (Fig. 1L). The lower |b*/ a*| values for T_4 and higher values for T_5 in the tea infusion indicated that the T_4 had a greener tint, while T_5 had a more yellowish appearance (Fig. 1G-I, K). In summary, among the samples subjected to different single-temperature drying settings, T_4 exhibited superior color quality, whereas T_5 displayed inferior color characteristics. In other words, Tencha dried at either excessively high or low single- temperature result in color quality that was suboptimal.

#### 3.1.2 Catechins and caffeine in tea infusion based on targeted analysis

**Fig. 2:**
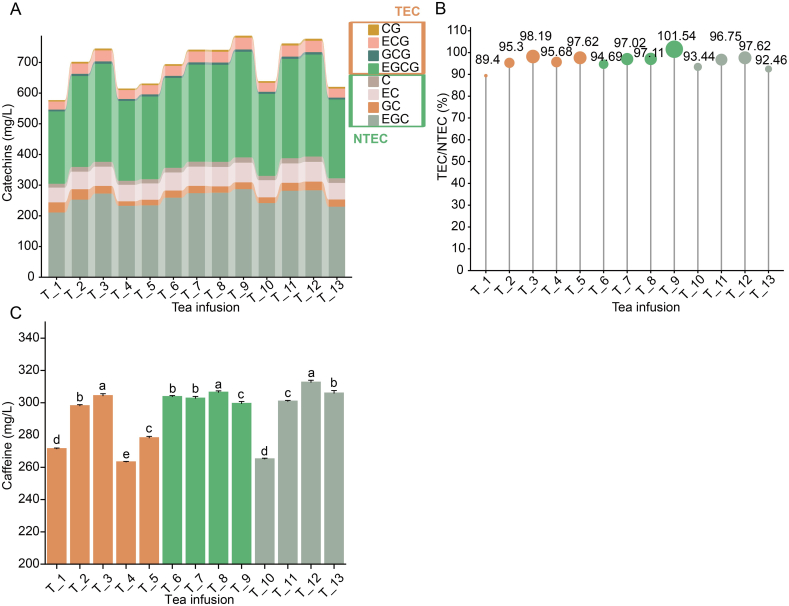
Changes in the catechins and caffeine in tea infusion under different drying settings temperatures. **A).** Concentrations of catechins. **B).** Ratio of ester catechins (TEC) to non-ester catechins (NTEC). The ester catechins were CG, ECG, GCG and EGCG and the non-ester catechins were C, EC, GC and EGC. **C).** Concentrations of caffeine. Catechin gallate, CG; epicatechin gallate, ECG; gallocatechin gallate, GCG; epigallocatechin gallate, EGCG; catechin, C; epicatechin, EC; gallocatechin, GC; epigallocatechin, EGC; total ester catechins, TEC; total non-ester catechins, NTEC.

During tea processing, the structure and content of major phenolic components, such as EGC, EC, EGCG, and ECG, were significantly influenced by factors like temperature, humidity. These effects are primarily due to the oxidation and degradation of catechins, which can occur during prolonged exposure to elevated temperatures (Victoria, Ananingsih, & Zhou, 2013). Different drying procedures for Tencha resulted in different rates of water loss, which in turn led to variations in catechin content (Fig. 2A). Among the samples, T_3 exhibited the highest catechin content at 745.29 mg/L, while T_1 had the lowest at 577.08 mg/L, with T_4 following at 614.94 mg/L. The ratio of ester to non-ester catechins was greater in samples T_3 and T_5. In contrast, sample T_4 demonstrated a more balanced ester to non-ester catechins ratio but a lower total catechin content (Fig. 2B). This result suggested that lower drying temperatures slowed down the rate of degradation and isomerization reactions of catechins. Caffeine in tea is prone to sublimation or interaction with catechins during the drying process of Tencha, impacting the flavor profiles of the tea. Significant differences in caffeine content were observed among tea samples processed under various drying temperature settings (*p* ≤ 0.5, Fig. 2C). Sample T_3 had the highest caffeine content, approximately 300 mg/L, whereas samples T_4 had the lowest, at around 87 % of T_3's caffeine content.

In relation to color attributes, T_3 had the highest total catechin content, paired with the lowest L* value (96.47), a* value (−3.87), and the highest b* value (13.12). Conversely, sample T_4 exhibited the highest L* value (96.81), a* value (−3.28), and the lowest b* value (10.98) (Fig. 1G-I & 2A). Researchers have linked these observable color changes to the presence of catechin oxidation products with larger molecular weights, such as theaflavins, thearubigins, and theabrownins (Bailey, Nursten, & McDowell, 1992; Gong, Zhang, Peng, Fan, & Dong, 2012; Li, Taylor, Ferruzzi, & Mauer, 2013; Sang, Lee, Hou, Ho, & Yang, 2005). Furthermore, EGCG could degraded into several key coloring substances due to molecular rearrangements under high-temperature conditions (Wang et al., 2022). Therefore, it was speculated that the observed color difference in Tencha may be attributed to the formation of these catechin oxidation products.

#### 3.1.3 Pigments in Tencha

**Fig. 3:**
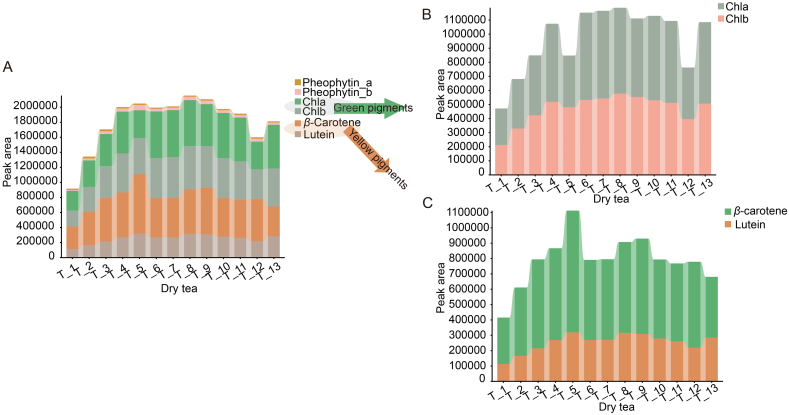
Changes in pigments in tencha under under different drying settings temperatures. **A).** Key tea pigments including lutein, *β*-carotene, Chlb, Chla, pheophytin_b, pheophytin_a). **B—C).** Green pigments (B, Chlb and Chla) and yellow pigments (C, lutein and *β*-carotene). (For interpretation of the references to color in this figure legend, the reader is referred to the web version of this article.)

Lipid-soluble pigments, primarily chlorophyll and carotenoids, are crucial for the color characteristics of tea (Wan, 2003). In this study, the contents of chlorophyll (Chla, Chlb, Phe-a, Phe-b) and carotenoids (lutein, *β*-carotene) in Tencha samples subjected to different drying procedures were analyzed. As summarized in Fig. 3A, lutein, *β*-carotene, Chla, Chlb were the predominant pigments, while Phe-a and Phe-b were present in lower amounts. Notably, the content of lutein and *β*-carotene in sample T_5 was significantly higher compared to other samples (Fig. 3C), probably due to the lower drying temperature (90 °C) which had a less destructive effect on carotenoids (Antonio, Patricia, & Delia, 2023). The data showed an increasing trend in the content of lutein and *β*-carotene from sample T_1 to T_5, indicating that lower drying temperatures were associated with higher levels of these carotenoids. Conversely, the content of Chla and Chlb, as well as the total pigments content, initially increased with decreasing drying temperature but then decreased when the temperature was reduced further, as observed from samples T_1 to T_5 (Fig. 3A-B). This may be related to the increased oxygen and humidity in the samples after lowering the temperature, which accelerated the degradation of chlorophyll. The specific reasons for this observed trend warrant further investigation.

Lutein and *β*-carotene are yellow pigments, while Chla and Chlb are green pigments. The relative contents of yellow and green pigments were compared among samples processed under different drying conditions (Fig. 3B-C). Sample T_1 exhibited the lowest levels of both yellow and green pigment, probably due to extensive degradation of chloroplast structure and chlorophyll release resulting from prolonged high-temperature drying ((130 °C). This high temperature also caused degradation of carotenoids, leading to weakened green and yellow coloration. Interestingly, sample T_4 contained the highest levels of green pigments and had the smallest ratio of yellow to green pigments, indicating a greener hue. In contrast, sample T_5 had a much higher content of yellow pigments relative to green pigments, resulting in a more yellow appearance. Overall, these findings suggested that a lower the drying temperature (e.g., T_4) yielded better color quality. However, below a certain temperature threshold (e.g., T_5), color quality deteriorated.

### 3.2 Color characteristics of Tencha using two-stage-temperature drying procedure

#### 3.2.1 Color sensory quality and chromatic difference analysis

A two-stage temperature drying procedure was applied to Tencha samples T_6, T_7, T_8, and T_9. This method involved an initial drying phase at varying high temperatures, followed by a secondary drying stage at 90 °C until complete desiccation was achieved (Table S1). Sensory evaluation revealed minimal differences among the four samples (Fig. 1A-C). Visually, Samples T_6 and T_7 presented a more vibrant green hue, garnering higher ratings of 32 points each. Additionally, the tea infusions from these samples displayed notably greater brightness, with scores of 13 and 14, respectively. Sample T_7 had a slight edge in overall sensory evaluation (Fig. 1C). Therefore, a relatively higher initial drying temperature yielded superior color quality of Tencha. The chromatic difference, indicated by the |b*/a*| value, showed an increasing trend from samples T_6 to T_9, as depicted in Fig. 1J-K. This trend suggested a reduction in the greenness of the samples and their corresponding infusions, indicating that samples T_6 and T_7 retained more green color compared to the others. The above results indicated that within the two-stage temperature drying procedure, Tencha samples with lower the initial drying temperature were less conducive to producing high color quality.

#### 3.2.2 Catechins and caffeine in tea infusion based on targeted analysis

The analysis of catechin content in Tencha sample T_6-T_9, processed using different drying procedure, indicated that sample T_9 exhibited the highest total catechin content and the highest ratio of ester to non-ester catechins (Fig. 2A-B). This result revealed that lower temperature (100 °C) favored the retention of more catechins in the samples. This result revealed that lower temperature (100 °C) favored the retention of more catechins in the samples. This result suggested that lower temperatures favor the retention of more catechins in the samples. In contrast, sample T_6 had significantly lower levels of individual catechin content (except for GC), total catechin content, and the ester to non-ester catechin ratio compared to samples T_7, T_8, and T_9. This resulted in a brighter color in the infusion of sample T_6. The caffeine content across the four samples were relatively consistent, with all samples showing levels around 300 mg/L (Fig. 2C). Despite this similarity in caffeine content, sample T_9, which had the higher concentration of major phenolic compounds, also exhibited poorer color quality, as indicated by its lower color score (Fig. 1).

#### 3.2.3 Pigments in Tencha

The relative contents of six pigments in sample T_6, T_7, T_8, and T_9 were analyzed, as shown in Fig. 3A-C. The analysis confirmed that samples T_6 and T_7 had lower contents of lutein and *β*-carotene, but higher levels of Chla. Conversely, sample T_9 had the lowest Chla content and higher *β*-carotene content. This may be due to the varying sensitivity of these substances to temperature. For example, lutein and *β*-carotene were more sensitive to high temperatures (120 and 130 °C) and Chla was more sensitive to low temperatures (100 °C). This may be due to the varying sensitivity of these substances to temperature. For example, lutein and *β*-carotene were more sensitive to high temperatures (120 and 130 °C) and Chla was more sensitive to low temperatures (100 °C). The green pigment contents in samples T_6, T_7, and T_8 were significantly higher (Fig. 3B), while the yellow pigment contents in T_6 and T_7 were significantly lower (Fig. 3C). This may be due to the varying sensitivity of these substances to temperature. For example, lutein and *β*-carotene were more sensitive to high temperatures and Chla was more sensitive to low temperatures. These findings indicated that the optimal drying procedure for achieving a greener Tencha involved an initial drying at 120/130 °C for 5 min, followed by drying at 90 °C until complete desiccation.

That is, in the two-stage temperature drying process, dried at 120/130 °C for 5 min, and then at 90 °C until fully dried was the optimal drying procedure. This procedure aligned with the color quality results described previously. In contrast, drying at a lower initial temperature (e.g., 100/110 °C for 10 min, followed by 90 °C until fully dried) resulted in a yellower Tencha. In conclusion, for the two-stage drying temperature setting, higher initial temperature setting (e.g., T_6 and T_7) positively impacted the color quality of Tencha, whereas lower initial temperature (e.g., T_9) was detrimental to achieving optimal color quality.

### 3.3 Color characteristics of Tencha using multiple-temperature drying procedure

#### 3.3.1 Color sensory quality and chromatic difference analysis

Tencha samples T_10, T_11, T_12 and T_13 were produced using drying procedure with multiple temperature settings (Table S1). Sensory evaluation revealed variation in color quality among these samples (Fig. 1A-C). Samples T_10 and T_13 exhibited superior color quality, characterized by enhanced greenness and brightness in their brewed tea infusions. Conversely, sample T_12, which underwent a gradual-rising temperature drying procedure, achieved the lowest overall color score of 41. Evidently, that drying method that involved temperature fluctuations was not appropriate for processing Tencha. The chromatic difference, represented by the |b*/a*| value, were lower for Tencha and its infusion in samples T_10 and T_13 (Fig. 1J-K), indicating a better greening effect and confirming the sensory evaluation results. In contrast, the |b*/a*| values for the T_12 sample of Tencha (5.30) and its infusion (3.46) were significantly higher (*p* ≤ 0.5), indicating a yellower color. These findings suggested that multiple temperature setting treatments had a significant impact on the color quality of Tencha, with variations in the temperature settings affecting the final color attributes of the tea.

#### 3.3.2 Catechins and caffeine in tea infusion based on targeted analysis

To monitor the phenolic changes resulting from various drying procedures, the contents of eight major phenolic compounds were compared among Tencha samples T_10, T_11, T_12 and T_13, which were processed using different multiple temperature settings (Fig. 2A-C). Sample T_12 exhibited the highest total catechin content of 777.22 mg/L and largest ratio of ester to non-ester catechins of 97.62 %. This elevated catechin content may contribute to the dull color and bitter taste of its tea infusion, as noted by Yu et al. (2023). In contrast, samples T_10 and T_13 had lower total catechin contents and a lower ratio of ester to non-ester catechins. This may be due to the higher initial temperature which promoted the degradation and transformation of catechins in the samples. Regarding caffeine content, sample T_10 had a significant lower caffeine concentration at approximately 265 mg/L, while sample T_12 had a significant higher caffeine concentration at about 312 mg/L (*p* ≤ 0.5, Fig. 2C). Combining the color difference and sensory data, it was evident that higher levels of polyphenols, as seen in sample T_12, adversely affected the color quality of Tencha.

#### 3.3.3 Pigments in Tencha

Fig. 3 contained the analysis results of pigments in Tencha samples processed using different multiple temperature setting. The analysis revealed that sample T_10, T_11 and T_13 had the highest contents of Chla and Chlb. In contrast, the chlorophyll degradation products, pheophytin a (Phe-a) and pheophytin b (Phe-b), were least abundant in sample T_13 and most abundant in sample T_12. Additionally, sample T_12 exhibited higher *β*-carotene content. As shown in Fig. 3B-C, sample T_10 had the highest content of green pigments and lowest ratio of yellow pigment to green pigment. Sample T_13 also had a low yellow-to-green pigment ratio, indicating that both T_10 and T_13 had greener colors. Conversely, sample T_12 had a higher yellow pigment content compared to green pigments, resulting in a more yellowish color. These results suggested that Tencha dried with a low temperature followed by a high temperature in the multiple temperature setting treatment tended to show more yellowing, which was detrimental to the color quality of Tencha.

Comparative analysis of single-stage, two-stage, and multiple temperature setting samples indicated that Tencha processed using two-stage or multiple temperature settings (samples T_6-T_13) exhibited better color quality compared to those processed using a single temperature setting (samples T_1-T_5). Specifically, Tencha dried at low temperatures (e.g., samples T_5, T_8, T_9) or with a gradual-rising temperature (e.g., sample T_12) showed poorer color quality, with a more pronounced yellow hue. In contrast, Tencha subjected to a short period of high-temperature drying followed by low-temperature drying (e.g., samples T_6, T_7, T_10, T_13) exhibited superior color quality. These findings were consistent with observations reported by Zhong et al. (2004).

### 3.4 Chlorophylls derivatives associated with the color of Tencha based on non-targeted analysis

**Fig. 4:**
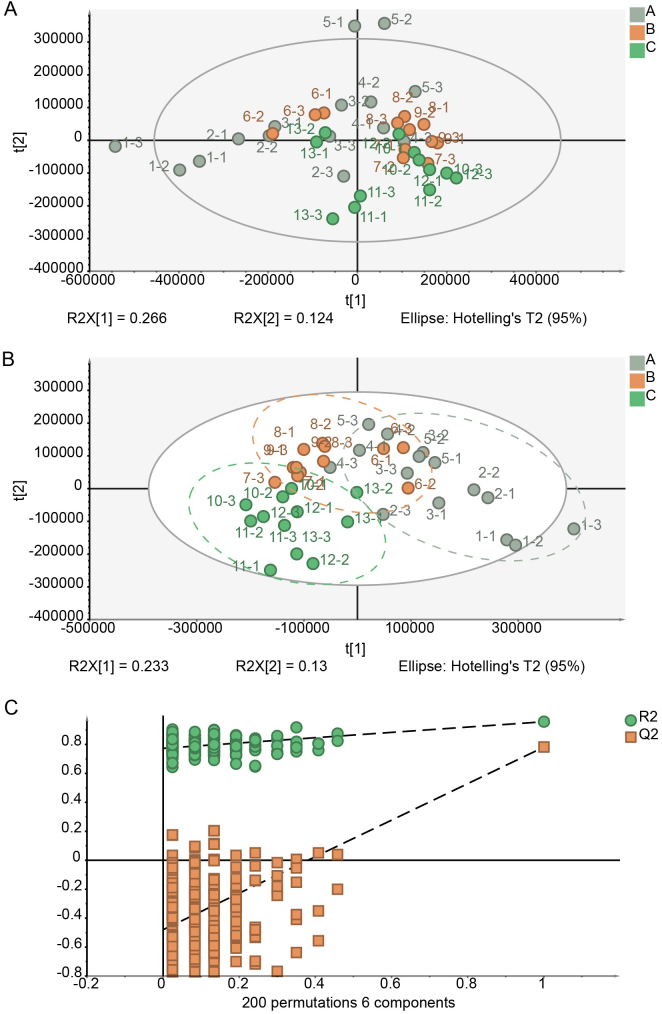
Chemometric analysis of Tencha under different drying settings temperatures. **A).** PCA. **B).** PLS-DA (R^2^X(cum) = 0.624, R^2^Y(cum) = 0.914, Q^2^(cum) = 0.619)). **C).** 200 permutations of PLS-DA. Legend A represented a single temperature setting program group, i.e., T_1-T_5; Legend B represented a two-stage temperature setting program group, i.e., T_6-T_9; Legend C represented a multi-stage temperature setting program group, i.e., T_10-T_13.

A non-targeted metabolic analysis was conducted on 13 Tencha samples processed under different drying temperatures and times. PCA, an unsupervised chemometric tool, was employed to provide a global overview of metabolic phenotype changes. The PCA score plot did not reveal significant separation among the different drying treatment groups (Fig. 4A). In contrast, the subsequent supervised PLS-DA score plot showed more pronounced separation among the groups (Fig. 4B), with no evidence of overfitting, as indicated by the model parameters (R^2^X (cum) = 0.624, R^2^Y (cum) = 0.914, Q^2^ (cum) = 0.619; Fig. 4C). This suggested that the drying treatments significantly influenced the chemical composition of Tencha. For further investigation, substances with Variable Importance in the Projection (VIP) values >1 and *p* values <0.05 were selected for identification by using the online databases (http://www.hmdb.ca/, http://www.mzcloud.org, http://www.chemspider.com/). A detailed list of chlorophyll and its derivatives identified in this analysis was displayed in Table 1.

**Table 1:** Key chlorophyll derivatives identified by non-targeted analysis based on UHPLC-HRMS.

No.	VIP (>1.0)	Compounds	Formula	Measured (*m/z*)#	RT (min)	*P*
1	5.302	Pheophytin a	C_55_H_74_N_4_O_5_	870.5645	12.301	<0.01
2	4.312	Chlorophyll b	C_55_H_70_MgN_4_O_6_	906.5136	9.644	<0.01
3	3.990	Pyropheophytin a	C_53_H_72_N_4_O_3_	812.5589	13.158	<0.01
4	2.995	Pheophytin b	C_55_H_72_N_4_O_6_	884.5440	10.905	<0.01
5	2.928	Chlorophyll a	C_55_H_72_MgN_4_O_5_	892.5341	11.123	<0.01
6	1.925	Chlorophyll b’ (s-epimer of Chlorophyll b)	C_55_H_70_MgN_4_O_6_	906.5135	10.071	<0.01
7	1.182	Pyropheophytin b	C_53_H_70_N_4_O_4_	826.5384	11.906	<0.01

# The samples were detected in positive ion mode (ESI^+^).

**Fig. 5:**
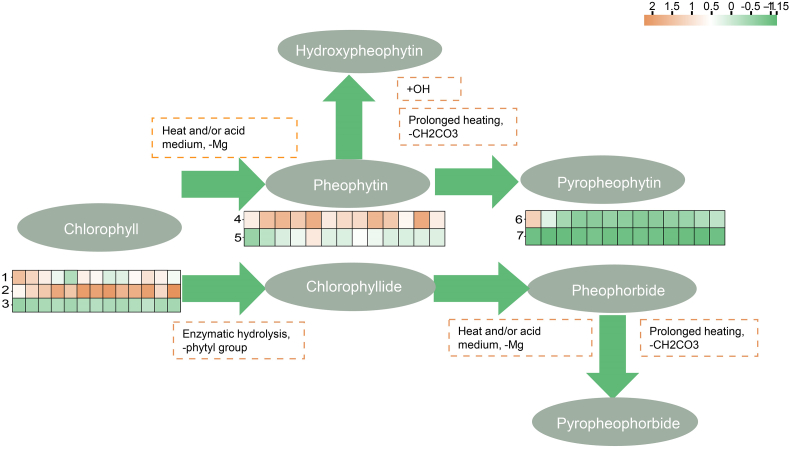
Formation mechanism of key chlorophyll derivatives in Tencha. The abscissa of the heat map involved was set to different drying temperature program settings, i.e., T_1-T_13, and the ordinate was set to different chlorophyll derivatives. The normalized scale was −1.15-2 (upper right corner). Below the “Chlorophyll” were the heat map changes of chlorophyll a (1), chlorophyll *b* (2), chlorophyll *b*’(s-epimer of chlorophyll *b*, 3) respectively, below the “Pheophytin” were the heat map changes of pheophytin a (4), pheophytin b (5) respectively and below the “Pyropheophytin” were the heat map changes of pyropheophytin a (6), pyropheophytin b (7) respectively.

The contents of chlorophylls derivatives in Tencha processed under different drying conditions was analyzed and compared (Fig. 5). Notably, chlorophyll *b* content as higher in sample T_6, T_7, T_10 and T_13 compared to T_1, T_5, and T_12. Conversely, pheophytin a (Phe-a) content was higher in samples T_5 and T_12. These results suggested that samples T_6, T_7, T_10, and T_13 retained more chlorophyll, whereas samples T_5 and T_12 experienced greater transformation of chlorophyll into various metabolites. This finding aligned with known chlorophyll metabolic pathways. As illustrated in Fig. 5, during tea drying, chlorophyll can lose its magnesium ion under heat, resulting in the formation of pheophytin. Prolonged heating can further remove the methyl group from pheophytin, producing pyropheophytin (Adhamatika, Murtini, & Sunarharum, 2021; Putri, Murtini, & Sunarharum, 2021). Additionally, chlorophyll may lose its phytyl group due to the action of chlorophyllase, leading to the formation of chlorophyllide, which can then be further degraded into pheophorbide and other products (Antoni et al., 2018). Chlorophyllase was typically activated at temperatures between 60 °C and 82 °C but degraded at high temperatures (> 100 °C) (Yilmaz & Gökmen, 2016). During the Tencha fixing stage, high-temperature steam at 100 °C rapidly inactivated chlorophyllase in the tea leaves. Therefore, while enzymatic degradation of chlorophyll occurred, its effect was less pronounced compared to the thermal degradation of chlorophyll, which played a more significant role. Chlorophyll imparted a bright green color, whereas its degradation products, such as pheophytin, presented a darker olive-brown hue (Li et al., 2021). As a result of the drying process, tea leaves transitioned from dark green to yellow-green, with chlorophyll degradation contributing to this color change (Li et al., 2023). The degree of chlorophyll degradation in Tencha varied significantly with different drying temperatures and times, leading to variations in the content of chlorophyll *a*nd its derivatives. These differences in chlorophyll derivatives collectively influenced the green color of Tencha.

Monitoring chlorophyll content during the processing of Tencha (the raw ingredient for Matcha) is crucial for determining the final color and quality of Matcha, as green color is a key indicator of high-quality Tencha. Therefore, optimizing the drying procedure to minimize chlorophyll degradation is essential for preserving the desirable green color in Tencha.

## 4 Conclusion

To summarize, drying treatment had a significant effect on the color quality of Tencha. Compared with single drying temperature drying, employing two or more temperature stages during the drying process better preserves Tencha's color quality. Specifically, Tencha dried at low temperatures (e.g., sample T_9) or using gradually increasing temperatures (e.g., sample T_12) exhibited inferior color quality, characterized by yellowish hues in both the leaves and tea infusion. In contrast, Tencha subjected to short-term high-temperature drying followed by low-temperature drying (e.g., samples T_6, T_7, T_10, and T_13) demonstrated superior color quality, with higher chlorophyll content and lower levels of lutein and *β*-carotene. The content of chlorophyll and its derivatives was identified as a critical factor affecting the color of Tencha. Therefore, the approach of initially drying Tencha at short-term high temperatures for a short duration, followed by a low-temperature drying phase, effectively preserved its green color. This method provided a valuable strategy for tea producers aiming to enhance the color quality of Tencha.

## Ethics statement

Prior to conducting the sensory tests covered in this manuscript, members of the panel at the Tea Research Institute of the Chinese Academy of Agricultural Sciences (Hangzhou, China) were informed of the content, requirements, and risks of the tests and signed the relevant informed consent forms. In addition, permission to conduct the sensory test was obtained from the institution.

## CRediT authorship contribution statement

**Ya-Lin Mao:** Writing – original draft, Methodology, Investigation, Data curation. **Jie-Qiong Wang:** Writing – review & editing. **Fang Wang:** Validation. **Qing-Qing Cao:** Writing – review & editing. **Jun-Feng Yin:** Supervision. **Yong-Quan Xu:** Writing – review & editing, Project administration, Funding acquisition.

## Declaration of competing interest

The authors declare that they have no known competing financial interests or personal relationships that could have appeared to influence the work reported in this paper.

## Data Availability

Data will be made available on request.

## Appendix A Supplementary data

Supplementary Table S1. Different drying temperature settings and series numbers for Tencha samples. Image 1

## References

[bb0005] Adhamatika A., Murtini E.S., Sunarharum W.B. (2021). The effect of leaf age and drying method on physico-chemical characteristics of pandan (*Pandanus amaryllifolius* Roxb.) leaves powder. IOP Conference Series: Earth and Environmental Science.

[bb0010] Antoni D.R., Diana C., Jordi E., Francesca V., Mercè B., Ramon C.G. (2018). A fast and reliable ultrahigh-performance liquid chromatography method to assess the fate of chlorophylls in teas and processed vegetable foodstuff. Journal of Chromatography A.

[bb0015] Antonio J., Patricia E., Delia B. (2023). Comprehensive review on carotenoid composition: Transformations during processing and storage of foods. Food Research International.

[bb0020] Bailey R.G., Nursten H.E., McDowell I. (1992). Isolation and analysis of a polymeric thearubigin fraction from tea. Journal of the Science of Food and Agriculture.

[bb0025] Cao Q.Q., Chen G.S., Xu Y.Q., Yin J.F. (2020). Studies on the color change and internal mechanism of the Huangjinya fresh tea leaves during tea processing. Journal of Chinese Institute of Food Science and Technology.

[bb0030] Dai Q.Y., Xiao T., Zhu B., Hua Z.X. (2020). Effect of lipid soluble pigments on color of green tea infusion. Journal of Anhui Agricultural University.

[bb0035] Engelhardt U.H., Bahar I., Delker U. (2023). Food borne toxicants in coffee: Acrylamide and furan derivative content in Arabica and Robusta coffees with different roasting profiles and varying degrees of roast. Beverage Plant Research.

[bb0040] Fu Y.Q., Wang J.Q., Chen J.X., Wang F., Xu Y.Q. (2020). Effect of baking on the flavor stability of green tea beverages. Food Chemistry.

[bb0045] Gaur S., Shivhare U., Ahmed J. (2006). Degradation of chlorophyll during processing of green vegetables: A review. Stewart Postharvest Review.

[bb0050] Gong J., Zhang Q., Peng C., Fan J., Dong W. (2012). Curie-point pyrolysis-gas chromatography-mass spectroscopic analysis of theabrownins from fermented Zijuan tea. Journal of Analytical and Applied Pyrolysis.

[bb0055] Kim J., Kang J., Park S., Han H., Lee K., Kim A. (2020). Effect of storage temperature on the antioxidant activity and catechins stability of Matcha (camellia Sinensis). Food Science and Biotechnology.

[bb0060] Lee J., Hwang Y.-S., Kang I.-K., Choung M.-G. (2015). Lipophilic pigments differentially respond to drying methods in tea (*Camellia sinensis* L.) leaves. LWT - Food Science and Technology.

[bb0065] Lenka D., Jana B., Dominika B., Klaudia J. (2015). Effect of drying methods on content of some natural pigments in *Urtica Dioica* L*.* and *Melissa Officinalls* L. Journal of Microbiology, Biotechnology and Food Sciences.

[bb0070] Li J., Hua J., Yuan H., Deng Y., Zhou Q., Yang Y. (2021). Investigation on green tea lipids and their metabolic variations during manufacturing by nontargeted lipidomics. Food Chemistry.

[bb0075] Li J., Hua J., Zhou Q., Dong C., Wang J., Deng Y. (2017). Comprehensive lipidome-wide profiling reveals dynamic changes of tea lipids during manufacturing process of black tea. Journal of Agricultural and Food Chemistry.

[bb0080] Li J., Yuan H., Rong Y., Qian M.C., Liu F., Hua J. (2023). Lipid metabolic characteristics and marker compounds of ripened Pu-erh tea during pile fermentation revealed by LC-MS-based lipidomics. Food Chemistry.

[bib206] Li N., Taylor L.S., Ferruzzi M.G., Mauer L.J. (2013). Color and chemical stability of teapolyphenol (−)-epigallocatechin-3-gallate in solution and solid states. Food ResearchInternational.

[bb0085] Li X.L., Jin J.J., Sun C.J., Ye D.P., Liu Y.F. (2019). Simultaneous determination of six main types of lipid-soluble pigments in green tea by visible and near-infrared spectroscopy. Food Chemistry.

[bb0090] Liu L., Zareef M., Wang Z., Li H., Chen Q., Ouyang Q. (2023). Monitoring chlorophyll changes during Tencha processing using portable near-infrared spectroscopy. Food Chemistry.

[bb0095] Liu S., Rong Y., Chen Q., Ouyang Q. (2024). Colorimetric sensor array combined with chemometric methods for the assessment of aroma produced during the drying of tencha. Food Chemistry.

[bb0100] Mao Y.L., Wang F., Yin J.F., Xu Y.Q. (2020). Quality analysis of Tencha made from different tea cultivars. Journal of Tea Science.

[bb0105] Martina O., Bohuslava T., Ivan S., Jana P., Petra Č. (2014). Evaluation of significant pigment in green teas of different origin. Potravinarstvo.

[bb0110] Natthawuddhi D., Yukiharu O. (2019). The influence of processing conditions on catechin, caffeine and chlorophyll contents of green tea (*Camelia sinensis*) leaves and infusions. LWT - Food Science and Technology.

[bb0115] Putri D.A., Murtini E.S., Sunarharum W.B. (2021). The characteristicsof dried Suji (*Dracaena angustifolia* (medik.) Roxb.) leaves powder produced by different drying methods and temperatures. IOP Conference Series: Earth and Environmental Science.

[bb0120] Sahar R., Mehdi R., Sayed A.H.G. (2016). Evaluation of seven different drying treatments in respect to total flavonoid, phenolic, vitamin C content, chlorophyll, antioxidant activity and color of green tea (*Camellia sinensis* or *C. Assamica*) leaves. Journal of Food Science and Technology.

[bb0125] Sang S.M., Lee M.J., Hou Z., Ho C.T., Yang C.S. (2005). Stability of tea polyphenol (−)-epigallocatechin-3-gallate and formation of dimers and epimers under common experimental conditions. Journal of Agricultural and Food Chemistry.

[bb0130] Tan H.R., Lau H., Liu S.Q., Tan L.P., Sakumoto S., Lassabliere B. (2019). Characterisation of key odourants in Japanese green tea using gas chromatography-olfactometry and gas chromatography-mass spectrometry. LWT - Food Science and Technology.

[bb0135] Tomohito S., Hideki H., Akiko M., Yuhei H. (2018). Effect of shading intensity on morphological and color traits and on chemical components of new tea (*Camellia sinensis* L.) shoots under direct covering cultivation. Journal of the Science of Food and Agriculture.

[bb0140] Victoria K., Ananingsih A.S., Zhou W.B. (2013). Green tea catechins during food processing and storage: A review on stability and detection. Food Research International.

[bb0145] Wan X.C. (2003).

[bb0150] Wang J.-Q., Dai Z.-S., Gao Y., Wang F., Chen J.-X., Feng Z.-H. (2023). Untargeted metabolomics coupled with chemometrics for flavor analysis of dahongpao oolong tea beverages under different storage conditions. LWT - Food Science and Technology.

[bb0155] Wang J.-Q., Fu Y.-Q., Chen J.-X., Wang F., Feng Z.-H., Yin J.-F. (2022). Effects of baking treatment on the sensory quality and physicochemical properties of green tea with different processing methods. Food Chemistry.

[bb0160] Wang J.Q., Fu Y.Q., Granato D., Yu P.G., Yin J.F., Zeng L., Xu Y.Q. (2022). Study on the color effects of (−)-epigallocatechin-3-gallate under different pH and temperatures in a model beverage system. Food Control.

[bb0165] Wang J.-Q., Tang B.-M., Gao Y., Chen J.-X., Wang F., Yin J.-F. (2024). Impact of heat treatment on the flavor stability of Longjing green tea beverages: Metabolomic insights and sensory correlations. Food Research International.

[bb0170] Wang L.F., Park S.C., Chung J.O., Baik J.H., Park S.K. (2004). The compounds contributing to the greenness of green tea. Journal of Food Science.

[bb0175] Wang Z., Yin J., Xie F.C. (2019). Matcha and its application in food and daily chemical products. China Tea.

[bb0180] Xiao Z., Zhang W., Guo W., Zhang L., Huang A., Tao M. (2022). Determining the effects of tencha-ro drying on key volatile compounds in tencha (Camellia sinensis) through gas chromatography–mass spectrometry. Journal of Food Science.

[bb0185] Xiao Z.P., Tao M., Liu Z.Q. (2021). Effects of stem removal on physicochemical properties and sensory quality of tencha beverages (*Camellia sinensis; Chuanxiaoye*). Journal of Food Science.

[bb0190] Yilmaz C., Gökmen V. (2016).

[bb0195] Yu Y., Zhu X., Ouyang W., Chen M., Jiang Y., Wang J. (2023). Effects of electromagnetic roller-hot-air-steam triple-coupled fixation on reducing the bitterness and astringency and improving the flavor quality of green tea. Food Chemistry: X.

[bb0200] Zhong Y.F., Li Z.L., Zhou Z.K., Yuan L.Y., Hu X., Gao F.H. (2004). Dynamics of the fluctuation of moisture and chlorophyll in green tea in the process of constant temperature drying. Journal of Southwest Agricultural University.

[bb0205] Zhu W.F., Liu X.Y., Cheng X., Li Y.Y., Liu L.L. (2023). Shading effects revisited: Comparisons of spring and autumn shading treatments reveal a seasonal-dependent regulation on amino acids in tea leaves. Beverage Plant Research.

