# Supplementary material for: Effect of different drying temperature settings on the color characteristics of Tencha
Source: Food Chem X. 2024 Nov 5;24:101963. doi: 10.1016/j.fochx.2024.101963 (PMC11584762; doi:10.1016/j.fochx.2024.101963)
Supplement: Supplementary file 1 — Supplementary Table S1. Different drying temperature settings and series numbers for Tencha samples. [file mmc1.docx]

# Effect of different drying temperature settings on the color characteristics of Tencha

Ya-Lin Mao ^a, b, 1^, Jie-Qiong Wang ^b,1^, Fang Wang ^b^, Qing-Qing Cao ^b^,

Jun-Feng Yin ^b^, Yong-Quan Xu ^b *^

*^a^ Modern Agricultural institute, Jiaxing Vocational & Technical College, Jiaxing 314036, China.*

*^b^ Tea Research Institute Chinese Academy of Agricultural Sciences, Key Laboratory of Biology, Genetics and Breeding of Special Economic Animals and Plants, Ministry of Agriculture and Rural Affairs, Hangzhou 310008, China.*

**Corresponding Authors**

^*^Yong-Quan Xu, Tel: +86-0571-86650594. Fax: +86-571-86650056. Email: [yqx33@126.com](mailto:yqx33@126.com)

^1^ These authors contributed equally.

**Table S1.** Different drying temperature settings and series numbers for Tencha samples.

| Samples | Different drying procedures |
| --- | --- |
| T_1 | Dried at 130°C until fully dried. |
| T_2 | Dried at 120℃ until fully dried. |
| T_3 | Dried at 110℃ until fully dried. |
| T_4 | Dried at 100℃ until fully dried. |
| T_5 | Dried at 90℃ until fully dried. |
| T_6 | Dried at 130°C for 5 min, and then at 90°C until fully dried. |
| T_7 | Dried at 120°C for 5 min, and then at 90°C until fully dried. |
| T_8 | Dried at 110°C for 5 min, and then at 90°C until fully dried. |
| T_9 | Dried at 100°C for 10 min, and then at 90°C until fully dried. |
| T_10 | Dried at 130°C for 5 min, then at 110°C for 5 min, and finally at 90°C until fully dried. |
| T_11 | Dried at 120°C for 5 min, then at 100°C for 5 min, and finally at 90°C until fully dried. |
| T_12 | Dried at 90°C for 5 min, then at 100°C for 5 min, and finally at 120°C until fully dried. |
| T_13 | Drying at 150°C, 95°C, 75°C and 65°C order for 40 min in dry room of Tencha. |
